# Network‐dependent remote effects and local tau burden jointly shape neuronal dysfunction in Alzheimer's disease

**DOI:** 10.1002/alz.71914

**Published:** 2026-10-07

**Authors:** Lukas Frontzkowski, Sebastian Roemer‐Cassiano, Johannes Gnörich, Daniel Janowitz, Hannah Eyob, Katharina Probst, Dario Dhoqina, Anna Dewenter, Anna Steward, Zeyu Zhu, Madleen Klonowski, Fabian Hirsch, Davina Biel, Melina Paulus, Jonathan Vöglein, Sonja Schönecker, Boris‐Stephan Rauchmann, Carolin Kurz, Carolin Koriath, Jan Häckert, Sophia Stöcklein, Carla Palleis, Robert Perneczky, Johannes Levin, Katharina Buerger, Rudolf A. Werner, Günter Höglinger, Matthias Brendel, Nicolai Franzmeier

**Affiliations:** ^1^ Institute for Stroke and Dementia Research (ISD) LMU University Hospital, Ludwig‐Maximilians‐University (LMU) Munich Munich Germany; ^2^ Department of Nuclear Medicine LMU University Hospital, Ludwig‐Maximilians‐University (LMU) Munich Munich Germany; ^3^ Department of Neurology LMU University Hospital, Ludwig‐Maximilians‐University (LMU) Munich Munich Germany; ^4^ German Center for Neurodegenerative Diseases (DZNE) Munich Munich Germany; ^5^ Munich Cluster for Systems Neurology (SyNergy) Munich Germany; ^6^ Department of Psychiatry and Psychotherapy LMU University Hospital, Ludwig‐Maximilians‐University (LMU) Munich Munich Germany; ^7^ Department of Neuroradiology LMU University Hospital, Ludwig‐Maximilians‐University (LMU) Munich Munich Germany; ^8^ Department of Psychiatry, Psychotherapy and Psychosomatics, Medical Faculty University of Augsburg, BKH Augsburg Augsburg Germany; ^9^ Department of Radiology LMU University Hospital, Ludwig‐Maximilians‐University (LMU) Munich Munich Germany; ^10^ Ageing Epidemiology (AGE) Research Unit, School of Public Health Imperial College London London UK; ^11^ Sheffield Institute for Translational Neuroscience (SITraN) University of Sheffield Sheffield UK; ^12^ The Russell H. Morgan Department of Radiology and Radiological Sciences, Division of Nuclear Medicine Johns Hopkins School of Medicine Baltimore Maryland USA; ^13^ Department of Psychiatry and Neurochemistry, Mölndal and Gothenburg University of Gothenburg, The Sahlgrenska Academy, Institute of Neuroscience and Physiology Gothenburg Sweden

**Keywords:** Alzheimer's disease, neuronal dysfunction, perfusion, tau, tau positron emission tomography

## Abstract

**BACKGROUND:**

Tau pathology in Alzheimer's disease (AD) propagates along the brain's connectome, yet the mechanisms by which tau drives neuronal dysfunction remain unclear. We hypothesized that dysfunction primarily emerges in regions close to tau‐harboring epicenters.

**METHODS:**

One hundred thirty‐one amyloid‐positive individuals underwent dynamic [^18^F]PI‐2620 tau positron emission tomography. Kinetic modeling yielded distribution volume ratios (DVRs) and perfusion (R1). Tau epicenters were defined as top 10% DVR regions. Connectivity‐based distance to epicenters was derived from normative resting‐state functional connectivity.

**RESULTS:**

At the individual level, hypoperfusion was more closely related to distance from tau epicenters than to local tau deposition, particularly in early AD, consistent with diaschisis‐like disconnection. With advancing biological disease severity, local tau deposition increasingly converged with hypoperfusion. Critically, hypoperfusion mediated the relationship between tau accumulation and cognitive decline.

**DISCUSSION:**

Tau epicenters shape the brain‐wide pattern of neuronal dysfunction, while local tau burden determines the degree of damage driving cognitive decline.

## Highlights

- Connectivity‐based distance to tau epicenters predicted the spatial pattern of cerebral hypoperfusion more strongly than local tau deposition at the individual level.
- Remote, connectivity‐based effects predominated over local tau deposition in early disease, consistent with diaschisis‐like disconnection.
- The association between local tau deposition and hypoperfusion strengthened with advancing global tau burden.
- Spatial mismatch between hypoperfusion and tau deposition was most pronounced in parietal and temporal regions in early Alzheimer's disease (AD), shifting toward frontal and cingulate cortices at intermediate stages.
- A stage‐dependent transition from connectivity‐constrained to locally driven hypoperfusion implicates distinct pathophysiological processes underlying neuronal dysfunction across the AD severity spectrum.

## 1 BACKGROUND

Alzheimer's disease (AD) is characterized by the deposition of extracellular amyloid beta (Aβ) plaques and intracellular neurofibrillary tau tangles, along with neuronal loss and cognitive decline. While Aβ plaques accumulate in a diffuse, brain‐wide manner, *post mortem* and tau positron emission tomography (PET) studies have consistently shown that tau accumulation emerges in local epicenters and expands in a network‐like pattern by spreading across connected neurons.^1^, ^2^ Tau pathology generally emerges closer to the onset of clinical symptoms than Aβ and is considered the primary driver of neuronal dysfunction and neurodegeneration in AD.^2^, ^3^, ^4^ Consistent with this central pathophysiological role of tau in AD progression, converging evidence suggests that the spatial distribution of tau pathology—and not Aβ—closely mirrors the clinical phenotype and is strongly associated with the degree of cognitive decline.^5^, ^6^, ^7^, ^8^ Furthermore, common neuroimaging markers of neuronal dysfunction, such as fluorodeoxyglucose (FDG) PET or perfusion PET, show substantial spatial concordance between hypometabolism or hypoperfusion and tau burden, suggesting an important link between local tau deposition and subsequent impairment in neuronal functioning.^9^, ^10^, ^11^, ^12^ However, despite this clear relationship, hypometabolism and hypoperfusion can extend well beyond regions of overt tau accumulation indicating that neuronal dysfunction also manifests in brain regions that are not yet locally affected by tau pathology.^13^, ^14^, ^15^

This spatial mismatch may arise for several non‐mutually exclusive reasons. In part, it may be apparent rather than mechanistic, reflecting either the inability of current tau tracers to detect soluble oligomeric tau species or the presence of frequent non‐AD co‐pathologies, such as Lewy body or transactive response DNA binding protein 43 kDa (TDP‐43) pathology, that drive neurodegeneration independently of tau.^16^ Beyond these explanations, however, several studies have reported diaschisis‐like network effects in AD, characterized by dysfunction in anatomically or functionally connected but tau PET–negative regions, paralleling remote metabolic disturbances seen after focal stroke lesions.^14^, ^17^ This spatial mismatch between the deposition of primary pathology and neuronal dysfunction is particularly evident in the early stages of AD, when tau pathology remains confined to a limited set of epicenters, whereas functional impairments are already detectable in anatomically remote, tau‐negative areas.^16^, ^18^, ^19^, ^20^, ^21^

**RESEARCH IN CONTEXT**

1. **Systematic review**: Prior studies have demonstrated that tau pathology in Alzheimer's disease (AD) propagates along the brain's connectome, with neuronal dysfunction frequently extending beyond regions harboring overt tau deposition. However, the relative contribution of local versus remote effects of tau pathology on neuronal dysfunction across disease stages remains insufficiently characterized.
2. **Interpretation**: At the individual level, cerebral hypoperfusion was more consistently associated with connectivity‐based distance to tau epicenters than with local tau deposition, particularly in early AD, consistent with a diaschisis‐like mechanism. With advancing disease, a negative association between local tau deposition and hypoperfusion progressively emerged, with hypoperfusion partially mediating tau's association with cognitive decline.
3. **Future directions**: Longitudinal studies are warranted to delineate the temporal dynamics between tau pathology and neuronal dysfunction. Future investigations should account for co‐pathologies affecting perfusion, and determine whether connectivity‐informed perfusion markers hold potential as functional readouts for monitoring therapeutic efficacy of tau‐targeting interventions.

Multiple questions arise from these observations: (1) Do tau‐harboring regions induce downstream dysfunction in remote but connected brain regions, potentially via network‐level disconnection or diaschisis‐like processes? (2) Does the relative effect of remote tau pathology decrease and the effects from local tau pathology increase as a function of biological disease severity? (3) To what extent does neuronal dysfunction mediate the association between regional tau pathology and cognitive decline on a brain‐wide level? Disentangling these local and network‐level contributions is critical for understanding the mechanisms by which tau pathology disrupts brain function and cognition.

We addressed these questions in a cohort of 131 Aβ‐positive patients across the AD continuum who underwent fully dynamic 60‐minute [^18^F]PI‐2620 PET scanning and structural magnetic resonance imaging (MRI) at the Ludwig‐Maximilians‐University (LMU) University Hospital in Munich, Germany. Using a recently described one‐stop‐shop protocol, dynamic [^18^F]PI‐2620 PET images were processed to leverage kinetic modeling and compute (1) R1 as a perfusion parameter mirroring neuronal function, and (2) distribution volume ratio (DVR) reflecting tau deposition.^22^, ^23^, ^24^ This protocol enables the assessment of both tau deposition and perfusion during a single PET scan, facilitating a simultaneous quantification of two imaging parameters.^22^, ^24^, ^25^ To model remote effects of tau pathology, we computed connectivity‐based distance to tau PET epicenters, which was based on a previously established resting‐state functional connectivity template determined in 69 healthy controls of the Alzheimer's Disease Neuroimaging Initiative (ADNI) sample.^26^, ^27^

Using these data, our first aim was to integrate tau PET, perfusion PET, and functional connectivity to disentangle local and network‐driven effects of tau pathology on neuronal dysfunction across the AD continuum. Specifically, we investigated whether cerebral hypoperfusion patterns are more strongly associated with the extent of local tau deposition or with downstream effects from tau epicenters. Our second aim was to assess whether these spatial relationships vary according to the spatial extent of tau pathology, and to identify brain regions most commonly affected by a spatial mismatch between hypoperfusion and tau pathology. Finally, our third aim was to examine whether hypoperfusion, as a proxy for neuronal dysfunction, mediates the association between tau accumulation and cognitive impairment. By adopting a connectome‐informed framework, we aim to provide mechanistic insight into how tau accumulation leads to neuronal dysfunction and ultimately drives cognitive decline in individual patients with AD.

## 2 METHODS

### 2.1 Cohort

We included 131 patients with AD (Table 1), who underwent fully dynamic PET scans using the second‐generation tau PET radiotracer [^18^F]PI‐2620 at the Department of Nuclear Medicine at LMU Hospital Munich. Scans were eligible for inclusion only if they comprised the complete 0 to 60 minute acquisition window and showed no evidence of motion artifacts, as determined by visual inspection. In addition, structural T1‐ or T2‐weighted MRI were collected as part of the clinical routine. Prior to tau PET imaging, participants underwent comprehensive clinical evaluation (including cognitive testing using the Mini‐Mental‐State Examination [MMSE]) and received diagnoses at either the Institute for Stroke and Dementia Research or the Departments of Neurology and Psychiatry with subsequent follow‐up examinations. Additionally, a subset of participants underwent lumbar puncture for cerebrospinal fluid (CSF) analysis during their stay at LMU University Hospital, Munich. CSF biomarker levels, including Aβ42, Aβ40, and the Aβ42/Aβ40 ratio, were measured using the Innotest enzyme‐linked immunosorbent assay kit (Fujirebio Europe N.V.). The established cutoff for the Aβ42/Aβ40 ratio was set at > 5.5%, as per standardized diagnostic procedures at the partnering laboratory. Biomarker data from CSF and/or Aβ PET ([^18^F]florbetaben or [^18^F]flutemetamol) were used to confirm amyloid positivity as part of routine diagnostic AD workup. Patients with AD across the entire clinical continuum were included, ranging from cognitively normal with subjective cognitive decline (SCD) and mild cognitive impairment (MCI) to dementia. SCD was defined as having normal performance on the Consortium to Establish a Registry for Alzheimer's Disease Neuropsychological (CERAD‐NP Plus) cognitive test battery, while MCI was defined as showing abnormal performance on at least one subscale of the CERAD‐NP Plus battery with preserved activities of daily living. Dementia was defined based on impairment of multiple CERAD‐NP Plus subscales, with additional impairment in activities of daily living. All patients on the AD spectrum either had an abnormally reduced Aβ42/Aβ40 ratio or a positive Aβ PET based on visual read. Written informed consent was obtained from all participants and all procedures were conducted in accordance with the Declaration of Helsinki. The protocols of harmonized LMU Hospital studies ActiGlia, ICON, and ASTEROID were approved by the local ethics committee of the LMU Munich (application numbers 17‐569, 19‐022, 21‐1000, and 21‐0721) and the German radiation protection authorities (BfS application IDs: Z5−22464/2017–047‐K‐G, ZD 3‐22464/2022‐189‐G, ZD 3‐22464/2023‐042‐G).

**TABLE 1:** Mean distribution of age, sex and MMSE scores across the entire cohort and per subgroup.

Baseline characteristics	All patients	SCD Aβ+	MCI Aβ+	Dementia Aβ+
N	131	8	41	82
Male sex (%)	51.9%	62.5%	58.5%	47.6%
Age, mean (SD)	70.9 (8.7)	73.1 (3.1)	69.5 (8.9)	71.4 (8.9)
MMSE (SD)	22.8 (5.5)	29.1 (1.1)	26.1 (2.5)	20.5 (5.6)
T+ visual read	89.3%	50.0%	85.4%	95.1%

Abbreviations: Aβ, amyloid beta; MCI, mild cognitive impairment; MMSE, Mini‐Mental State Examination; SCD, subjective cognitive decline; SD, standard deviation.

### 2.2 PET imaging

[^18^F]PI‐2620 PET imaging in combination with computed tomography (CT) was performed in a full dynamic setting (minimum scan duration: 0–60 minutes p.i.) on two different scanners at the Department of Nuclear Medicine of the LMU Hospital using the established standard parameter for brain PET imaging as follows: PET data were acquired on a Siemens Biograph TruePoint 64 PET/CT or a Siemens mCT. The dynamic brain PET data were acquired in three‐dimensional list mode over 60 minutes, and reconstructed into a 336 × 336 × 109 matrix (voxel size: 1.02 × 1.02 × 2.03 mm^3^) using the built‐in ordered subset expectation maximization (OSEM) algorithm with 4 iterations, 21 subsets, and a 5 mm Gaussian filter. A low dose CT served for attenuation correction. Frame binning (*n* = 35) was standardized to 12 × 5 seconds, 6 × 10 seconds, 3 × 20 seconds, 7 × 60 seconds, 4 × 300 seconds, and 3 × 600 seconds.

### 2.3 PET quantitative assessment

Dynamic [^18^F]PI‐2620 PET scans and T1‐ or T2‐weighted structural MRI data were processed using an in‐house, fully automated PET quantification pipeline. First, motion correction of dynamic PET data was performed using the Fast Algorithm for Motion Correction (FALCON), enabling correction of both rigid and non‐linear motion artifacts.^28^ Motion‐corrected PET images were co‐registered to their corresponding structural MRI scan in native space. T1‐ or T2‐weighted MRI scans with low spatial resolution (i.e., > 1.5 mm isotropic voxels) were upsampled to a high‐resolution T1 image using the convolutional neural network (CNN) SynthSR implemented in FreeSurfer.^29^ The Computational Anatomy Toolbox 12 (CAT12) was used for tissue segmentation and spatial normalization of the final high‐resolution MRI data to Montreal Neurological Institute (MNI) space.^30^ The resulting inverted deformation fields were subsequently applied to the co‐registered dynamic PET images to achieve transformation into MNI space. Voxel‐wise R1 perfusion images (Figure 1) were generated using the simplified reference tissue model 2 (SRTM2), with the conventional inferior cerebellar gray matter (GM) serving as reference tissue.^23^ In addition, DVR images—reflecting overall tracer binding—were also calculated using the conventional reference region (Figure 1). No partial volume correction (PVC) was applied to the PET data, in line with our previous work, in which partial volume effects were not a relevant confounder of [^18^F]PI‐2620 R1 or DVR quantification.^31^ R1 and DVR *z* scores were computed relative to an in‐house dataset of 25 healthy control participants. Of note, late‐phase standardized uptake value ratio (SUVR) images were computed as an alternative quantification measure (Figure S1 in supporting information). Regional connectivity‐based distance to tau PET epicenters was assessed as previously reported.^26^, ^27^ In brief, DVR *z* score images were parcellated into 200 cortical regions of interest (ROIs) using the Schaefer cortical brain atlas.^32^ Specifically, subject‐ and ROI‐specific DVR *z* scores were concatenated in a 2D (two‐dimensional) matrix (subjects x ROIs), which was subsequently rank ordered by row sums and column sums. The tau spreading sequence was determined on the basis of the rank order of ROIs. These epicenters were defined both at the group level (top 10% of ROIs by mean DVR *z* score across the cohort) for group‐level analyses and, separately, within each individual patient (top 10% of that patient's own DVR *z* scores) for all subject‐specific analyses (Figure S2 in supporting information). Connectivity‐based distance to tau epicenters was determined on the basis of a functional connectivity template determined in 69 healthy controls of the ADNI sample with high‐resolution functional MRI.^26^, ^27^

**FIGURE 1:**
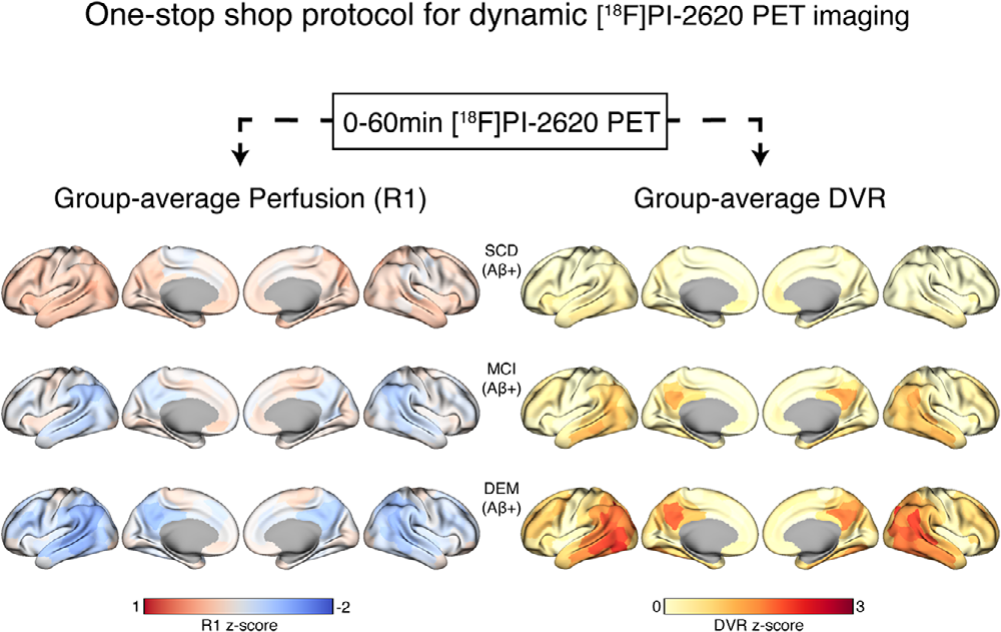
Group‐average [^18^F]PI‐2620 perfusion (R1) and DVR *z* score images rendered on cortical surfaces for each subgroup. Kinetic modeling was used to estimate parameters of perfusion (R1) and tau deposition (DVR). Inferior cerebellar GM served as reference tissue for both image modalities. DEM, dementia; DVR, distribution volume ratio; GM, gray matter; MCI, mild cognitive impairment; PET, positron emission tomography; SCD, subjective cognitive decline

### 2.4 Statistics

Statistical analyses were performed in Python (version 3.9.20) using Visual Studio Code. To address our first study aim, we assessed the group‐level spatial association between cerebral perfusion and (1) regional connectivity‐based distance to tau PET epicenters and (2) regional tau deposition (DVR) using linear regression, adjusted for age, sex, and PET scanner. To compare these spatial associations at the subject level, we fitted two separate single‐predictor linear regressions within each patient, across that patient's 200 cortical regions: perfusion on connectivity‐based distance to epicenters, and perfusion on local tau deposition. All variables were standardized (*z* scored) prior to fitting, so that the two resulting subject‐specific standardized β coefficients were directly comparable; tau epicenters were defined individually for each patient (the 10% of ROIs with that patient's highest DVR *z* scores). For each predictor, we then tested whether its subject‐level standardized β coefficients were reliably associated with perfusion across the cohort using a one‐sample *t* test against zero. In addition, the two subject‐level associations were directly compared using a paired *t* test across patients.

To address our second aim, we examined whether the strength of spatial associations derived from both models—(1) connectivity‐based distance to tau epicenters and cerebral hypoperfusion, and (2) between regional tau deposition and cerebral hypoperfusion—varied as a function of biological disease severity, operationalized by the global spatial extent of [^18^F]PI‐2620 tau PET (DVR). Specifically, global spatial extent was calculated as the proportion of tau PET–positive GM voxels (i.e., above a *z* score of 1.5) relative to the total number of GM brain voxels.^33^ To identify the most appropriate model specification, we compared linear, second‐order (quadratic), third‐order (cubic), and fourth‐order (quartic) models for each analysis using the Akaike information criterion (AIC) and Bayesian information criterion (BIC). To quantify the spatial tau‐perfusion mismatch, we counted, for each patient, the number of cortical regions that were hypoperfused (R1 *z* ← 1) but not tau positive (DVR *z* < 1) and tested its dependence on the global spatial extent of tau using a one‐way analysis of variance (ANOVA) across the three extent‐based tertile groups and a Spearman correlation across all patients.

For our third aim, linear regression models were fitted to examine associations among regional tau deposition, cerebral hypoperfusion, and cognitive performance as indexed by the MMSE, with all models adjusted for age, sex, and PET scanner. To account for multiple comparisons across 200 ROIs, results were corrected using false discovery rate (FDR) correction, with an alpha level of *P *< 0.05.

Finally, mediation analyses were performed to assess whether regional hypoperfusion may act as a mediator of the relationship between regional tau deposition and MMSE, using bootstrapping with 1000 resamples to estimate the indirect effect (average causal mediation effect [ACME]). Again, all analyses were corrected for age, sex, and PET scanner. Given the large number of ROIs (*n* = 200), results were corrected for multiple comparisons using FDR correction at a threshold of *P *< 0.05.

## 3 RESULTS

### 3.1 Cohort demographics

To cover the AD continuum, the sample encompassed eight patients with SCD, 41 with MCI, and 82 patients with AD dementia. Sample demographics and clinical characteristics can be found in Table 1.

### 3.2 Patterns of cerebral hypoperfusion are more strongly associated with connectivity‐based distance to tau epicenters than with local tau deposition

To address our first aim, we examined whether cerebral hypoperfusion—serving as a proxy for neuronal dysfunction—was spatially associated with (1) regional tau deposition and (2) connectivity‐based distance to tau epicenters. To this end, tau epicenters were defined as the top 10% of cortical regions exhibiting the highest tau PET DVR *z* scores. For each cortical ROI from the Schaefer 200 atlas, we calculated the mean connectivity‐based distance to tau epicenters.^32^ At the group level, we evaluated two linear regression models to assess (1) the association between cerebral perfusion and regional tau deposition, and (2) the association between cerebral perfusion and connectivity‐based distance to tau epicenters. Cerebral hypoperfusion was significantly associated with higher tau deposition (*R^2 ^* = 0.45, *P *< 0.001, Figure 2A) as well as with shorter connectivity‐based distance to tau epicenters (*R^2 ^* = 0.39, *P *< 0.001, Figure 2B). To compare these associations at the subject level, we fitted both spatial models separately within each patient and extracted the two subject‐specific standardized β coefficients. Across the 131 patients, cerebral perfusion was significantly associated with connectivity‐based distance to epicenters (mean β = 0.21, *P *< 10^−12^), whereas it showed no significant association with local tau deposition (mean β = 0.03, *P *= 0.43). At the individual level, directly comparing the two associations within patients confirmed that connectivity‐based distance was significantly more strongly associated with hypoperfusion than local tau deposition (*t*[130] = 2.81, *P *= 0.006, Figure 2C). All analyses were replicated using SUVR as an alternative tau quantification, for which connectivity‐based distance to epicenters again markedly exceeded local tau deposition in both the strength and consistency of its association with perfusion (Figure S3 in supporting information). Overall, these results suggest that spatial patterns of cerebral hypoperfusion are better captured by downstream effects from tau epicenters than by local effects from tau deposition.

**FIGURE 2:**
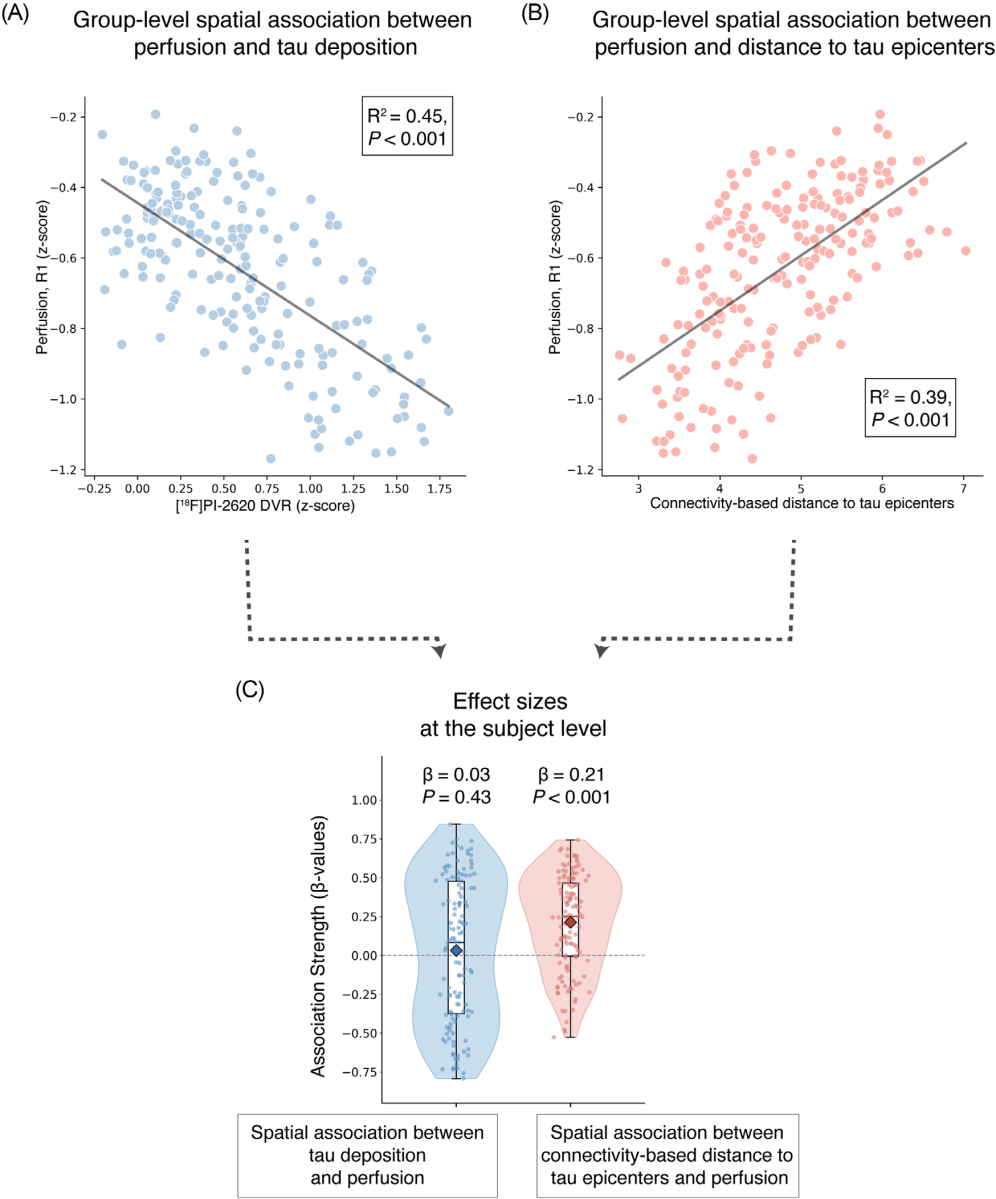
A, Scatterplot depicting the group‐level spatial association between cerebral perfusion (R1) and tau deposition (DVR) based on the Schaefer 200 cortical atlas. The conventional inferior cerebellar gray matter served as reference tissue. B, Group‐level spatial association between cerebral perfusion (R1) and connectivity‐based distance to tau epicenters as scatterplot. C, Distribution of the subject‐level standardized β coefficients quantifying the spatial association of cerebral perfusion with local tau deposition (pastel blue) and with connectivity‐based distance to tau epicenters (pastel pink) across all 131 patients. Each dot represents one patient; violins show the full distribution, boxes the median and interquartile range, and diamonds the group mean. The β and *P* values above each distribution denote the mean standardized β and the corresponding one‐sample *t* test against zero. Pastel blue, spatial association between tau deposition and perfusion. Pastel pink, spatial association between connectivity‐based distance to tau epicenters and perfusion. DVR, distribution volume ratio

### 3.3 Tau spread–dependent shift from remote to local tau‐related cerebral hypoperfusion across the AD continuum

To address our second aim, we leveraged the effect sizes derived from the subject‐specific spatial association models and tested their relationship with biological AD disease severity as operationalized by the global spatial extent of [^18^F]PI‐2620 tau PET DVR. Specifically, we tested multiple model approaches (i.e., linear, quadratic, cubic, and quartic) to identify the best‐fitting model for the data. For the spatial overlap between tau deposition and cerebral hypoperfusion, we observed that the spatial coupling between local tau deposition and hypoperfusion shifted continuously with higher global spatial extent of tau PET DVR, from a positive association at low tau extent to a robustly negative one at high extent (β = −0.35, *P *< 0.001, Figure 3A). In contrast, the coupling between perfusion and connectivity‐based distance to the epicenters did not decline as tau became more widespread. It showed no linear dependence on tau extent (β = 0.04, *P *= 0.672); instead, a quadratic model best captured the relationship, revealing a non‐monotonic, inverted U pattern in which the remote coupling was strongest at low‐to‐intermediate tau extent (first‐order β = 0.58, *P *= 0.046; second‐order β = −0.57, *P *= 0.048; Figure 3B). Among all models tested (linear, quadratic, cubic, quartic), the quadratic was the only one with significant terms and yielded the lowest AIC (374.3), with the BIC only marginally preferring the simpler linear model (387.9 vs. 388.7). The remote effect was thus sustained across the entire disease continuum rather than diminishing with advancing pathology. Given that the local tau–hypoperfusion association was confined to advanced tau extent (Figure 3A) whereas the remote effect persisted regardless of how widespread tau was (Figure 3B), hypoperfusion should be spatially separated from tau when pathology is still regionally confined and should increasingly coincide with tau as it spreads. To further test the relationship between the spatial dissociation of tau pathology and cerebral hypoperfusion, we used a tertile split to create three groups of patients with AD (i.e., Q1–Q3) based on the global spatial extent of [^18^F]PI‐2620 tau PET DVR.^33^ For each group, we used a region‐based mismatch approach to display the spatial tau–perfusion dissociation by summing the number of patients with AD who showed reduced perfusion (*z* score < –1.0) but not yet tau deposition (*z* score < 1.0) in a given ROI. Spatial pathology mismatch was most evident in patients with AD with low or medium spatial extent (Q1 and Q2), with a predominant localization in the parietal and temporal cortex in Q1, shifting toward frontal regions and the cingulum in Q2 (Figure 3C). In patients with AD with the highest global tau extent (Q3), spatial pathology mismatch was largely absent, reflecting a near complete overlap between tau deposition and cerebral hypoperfusion (Figure 3C). Statistically, the number of mismatch regions per patient decreased significantly with increasing global tau extent (standardized β = −0.22, *P *= 0.02; Spearman ρ = −0.32, *P *< 0.001) and differed across the tertile groups (one‐way ANOVA *P *< 0.001), being comparable in the low‐ and medium‐extent groups but markedly lower in the high‐extent group. Notably, all analyses were replicated using SUVR as an alternative quantification measure, yielding consistent results (Figure S4 in supporting information). Together, these results indicate a shift from remote to local effects across increasing levels of global tauopathy, with the spatial mismatch between tau deposition and cerebral hypoperfusion being most pronounced at earlier stages of biological disease severity.

**FIGURE 3:**
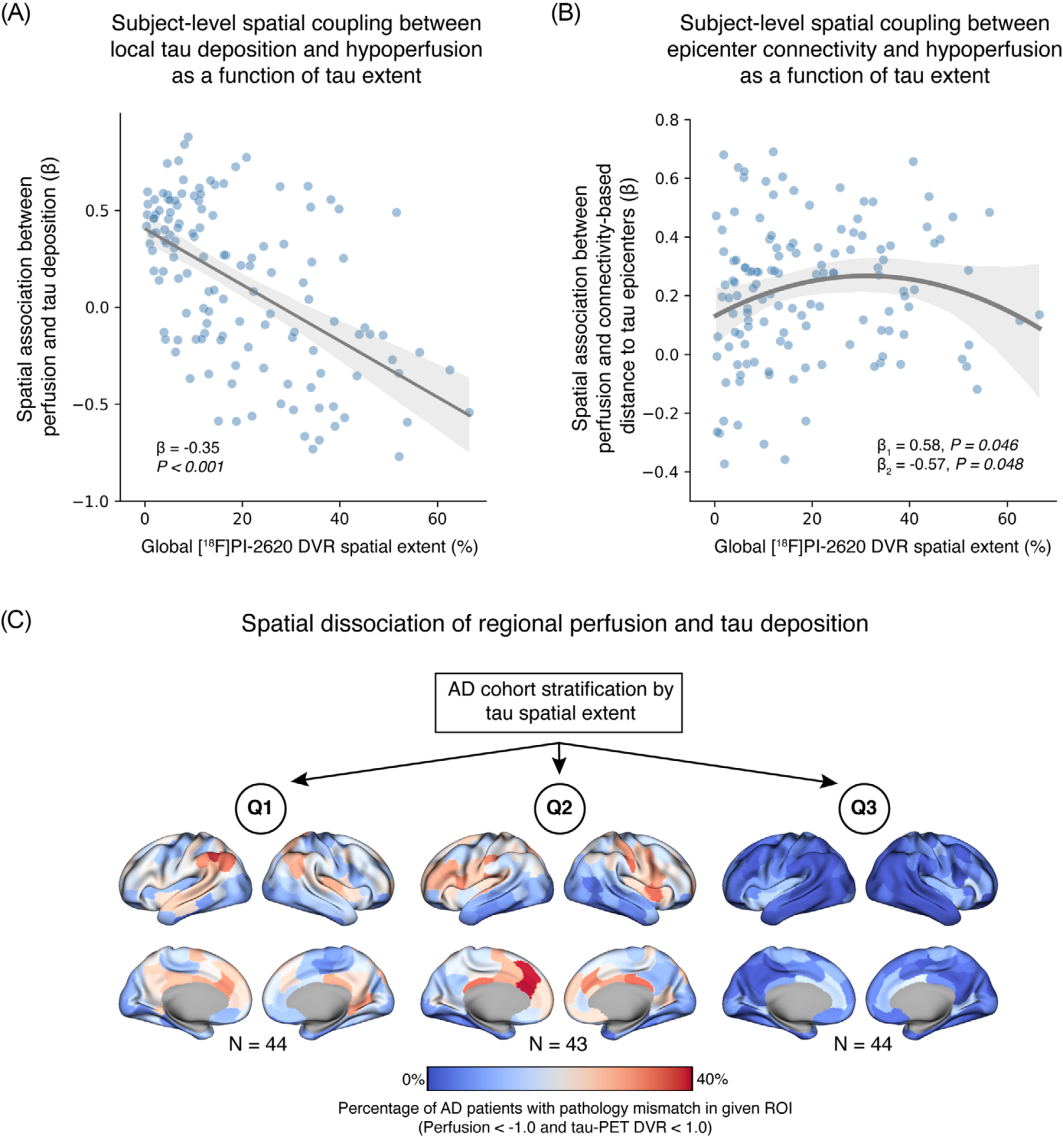
A, Scatterplot illustrating the individual effect sizes between tau deposition (DVR) and cerebral perfusion (R1) as a function of the global spatial extent of tau deposition (proportion of DVR positive—i.e., > 1.5) voxels relative to the total numbers of brain voxels). B, Scatterplot illustrating the individual effect sizes between connectivity‐based distance to tau epicenters and perfusion as a function of the global spatial extent of tau deposition. C, Spatial dissociation between regional hypoperfusion and tau deposition illustrated for three groups of patients with AD. Patients with AD were assigned to three groups based on the amount of spatial tau extent. Surface renderings illustrate the total number of patients with AD that show a perfusion–tau mismatch in a given ROI. Spatial pathology mismatch is higher in groups with low (Q1) or medium (Q2) spatial tau extent with a predominant localization in the parietal and temporal cortex in Q1, shifting toward frontal regions and the cingulum in Q2 (red). In patients with AD with the highest global tau extent (Q3), spatial mismatch was largely absent, reflecting a near complete overlap between tau deposition and cerebral perfusion (blue). Q1. low spatial extent of tau pathology; Q2, medium spatial extent of tau pathology, Q3, high spatial extent of tau pathology. AD, Alzheimer's disease; DVR, distribution volume ratio; PET, positron emission tomography; ROI, region of interest

### 3.4 Cerebral hypoperfusion mediates the association between tau deposition and cognitive decline

Our final aim was to determine whether cerebral perfusion mediates the relationship between tau deposition and cognitive decline in AD. We first examined the association between regional [^18^F]PI‐2620 DVR and global cognition as indexed by MMSE. This analysis revealed robust, widespread associations between stronger tau deposition and lower MMSE scores, predominantly in temporal, parietal, and occipital cortices (Figure 4A). We next assessed the relationship between regional hypoperfusion and MMSE, which yielded comparable brain‐wide spatial patterns of associations, with reduced perfusion similarly linked to lower MMSE scores (Figure 4B). Finally, to formally test whether hypoperfusion mediates the association between tau deposition and cognitive decline, we performed region‐wise mediation analyses. These models demonstrated that regional hypoperfusion partially mediates the effect of tau accumulation on MMSE scores primarily in temporal and parietal cortical regions (Figure 4C). All analyses were corrected for multiple testing and adjusted for age, sex, and PET scanner. Collectively, these findings suggest that cerebral hypoperfusion serves as a mechanistic link between regional tau deposition and cognitive decline, partially accounting for the detrimental effects of tau pathology on global cognition in AD (Figure 4D).

**FIGURE 4:**
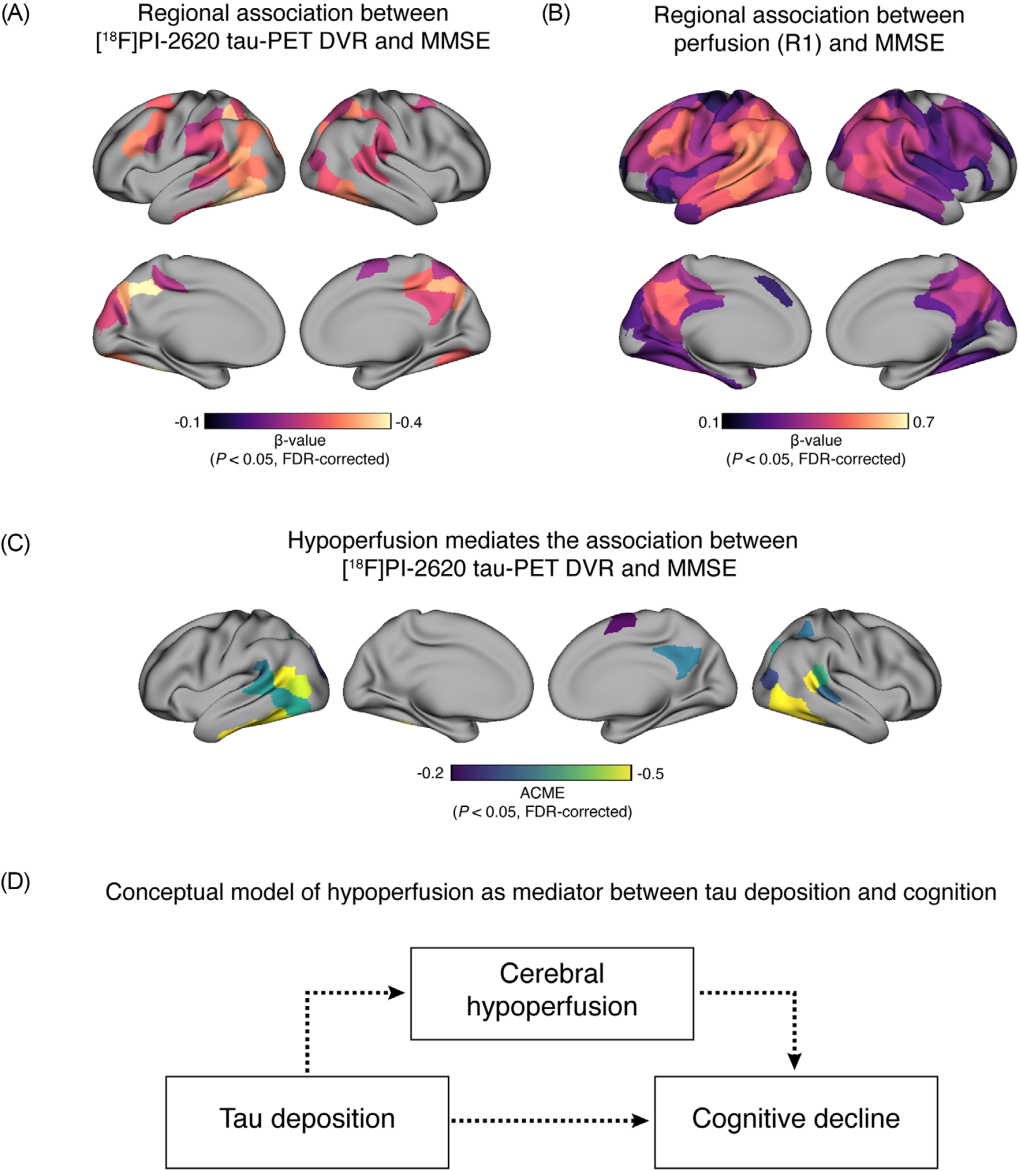
A, Surface renderings illustrating the regional association (β) between [^18^F]PI‐2620 tau PET DVR and MMSE. B, Surface renderings illustrating the regional association (β) between cerebral perfusion (R1) and MMSE. C, Surface renderings illustrating regional mediation results (ACME), illustrating the effect of tau pathology on MMSE through cerebral hypoperfusion. Brain regions of non‐significant association are depicted in gray. D, Diagram of the proposed pathophysiological model linking tau deposition to cognitive decline via cerebral hypoperfusion, with both indirect and direct effects considered. The depicted directionality reflects a hypothesis‐driven statistical model grounded in the established role of tau as an upstream molecular driver in AD; the arrows denote modeled statistical pathways and should not be interpreted as evidence of temporal precedence or causality. Given the cross‐sectional design, alternative orderings—including hypoperfusion preceding, or bidirectionally interacting with, tau—cannot be excluded. ACME, average causal mediation effect; AD, Alzheimer's disease; DVR, distribution volume ratio; FDR, false discovery rate; MMSE, Mini‐Mental State Examination; PET, positron emission tomography;

## 4 DISCUSSION

In the present study, we aimed to characterize local and remote effects of tau pathology on neuronal dysfunction across the AD continuum by leveraging a fully dynamic [^18^F]PI‐2620 scanning protocol to simultaneously quantify cerebral perfusion and tau deposition. Specifically, we investigated whether (1) spatial patterns of hypoperfusion are more strongly associated with local tau deposition or remote, diaschisis‐like effects from cortical tau epicenters; (2) the relative strength of these associations shifts as a function of biological AD disease severity; and (3) hypoperfusion mediates the relationship between tau deposition and cognitive decline on a brain‐wide level. By addressing these questions, we aimed to delineate the mechanisms by which tau pathology translates into distributed neuronal dysfunction and cognitive impairment across the AD continuum.

Our first main finding shows that the spatial pattern of cerebral hypoperfusion was more closely aligned to the proximity to cortical tau epicenters than local tau deposition on the individual level. Second, the relative contribution of local versus remote tau‐related effects shifted as a function of biological AD disease severity, with remote, connectivity‐based effects consistently present across the continuum whereas the negative association between local tau deposition and hypoperfusion progressively emerged at more advanced disease levels, suggesting stage‐dependent mechanisms of neuronal dysfunction. Third, cerebral hypoperfusion partially mediated the association between regional tau burden and MMSE, supporting a mechanistic link among tau accumulation, neuronal dysfunction, and cognitive decline.^34^

As a first main finding, we report that cerebral hypoperfusion is significantly associated not only with local tau deposition but also with remote effects from connected cortical tau epicenters. Importantly, at the individual level, the spatial association between cerebral hypoperfusion and connectivity‐based distance to tau epicenters was stronger than the association with local tau deposition. These findings suggest that neuronal dysfunction cannot be attributed to local effects of tau pathology alone but reflects potential tau‐related downstream effects.^13^, ^16^, ^17^, ^35^, ^36^ Consistent with this interpretation, multiple neuroimaging studies have shown that neuronal dysfunction in the posterior cingulate cortex relates to structural or functional disconnections from regions that are affected early in the course of AD.^35^, ^37^, ^38^ These observations are further supported by PET studies demonstrating remote effects of focal pathology on distant but anatomically connected brain areas, consistent with network‐based mechanisms such as crossed cerebellar diaschisis in stroke.^17^ Taken together, these findings support a disease model in which tau‐related neuronal dysfunction reflects distributed network disruption rather than purely local pathological burden. This interpretation is further strengthened by evidence showing that FDG PET–derived measures extend diagnostic performance of tau deposition, suggesting that including measures of neuronal dysfunction may better capture the impact of tau pathology than local tau deposition itself.^25^, ^39^

Another key finding of our study is that the relative impact of local and remote tau‐related effects shifted with increasing biological disease severity in AD. The spatial concordance between tau deposition and cerebral hypoperfusion increased linearly with the spatial extent of tau, indicating that a negative tau–hypoperfusion relationship progressively emerges, and local tau becomes a determinant of regional neuronal dysfunction in later disease stages. In contrast, the association between hypoperfusion and connectivity‐based distance to tau epicenters showed no clear dependence on the spatial extent of tau, indicating that remote effects were present across the continuum rather than diminishing as disease progressed. These findings suggest a stage‐dependent transition in mechanisms underlying neuronal dysfunction. In early AD, when tau pathology is spatially restricted, remote downstream effects appear to dominate.^16^ Hypoperfusion may therefore manifest in regions that are functionally connected to tau epicenters but not yet tau positive themselves, leading to a spatial mismatch between PET‐detectable molecular pathology and neuronal dysfunction. Indeed, arterial spin labeling (ASL) MRI studies revealed that hypoperfusion may precede tau pathology within the temporo‐spatial sequence of AD progression, particularly in vulnerable brain regions such as the entorhinal cortex.^15^ This interpretation is supported by our mismatch analysis, which revealed prominent cortical hypoperfusion without overt tau deposition in frontal and parietal regions—particularly within the cingulum and inferior frontal cortices—in patients with AD with low to medium spatial extent of tau.^35^ Notably, this mismatch decreased significantly as tau pathology became more widespread. Tau deposition and hypoperfusion thereby converged spatially, becoming increasingly concordant at more advanced stages.^40^ At advanced stages, neuronal dysfunction may thus be more directly driven by local neurotoxic effects of tau aggregates, which is consistent with a previous analysis of voxel‐wise‐correlations between hypometabolism and local atrophy in patients with AD dementia.^41^ Taken together, our findings indicate that tau pathology leads to early network‐mediated dysfunction and transitions into predominantly local pathology‐driven damage as the disease advances.

Furthermore, our mediation analyses revealed that cerebral hypoperfusion partially mediates the association between regional tau deposition and global cognitive deficits. Consistent with previous reports, both higher tau deposition and reduced perfusion were independently associated with lower MMSE scores, predominantly involving temporo‐parietal and occipital cortices.^34^, ^42^, ^43^ Importantly, a substantial proportion of the tau–cognition relationship was mediated by hypoperfusion, indicating that neuronal dysfunction constitutes a critical intermediate step linking molecular pathology to clinical decline.^44^ These findings are highly consistent with the notion that tau pathology acts as the primary driver of neurodegeneration and cognitive impairment in AD.^2^, ^3^, ^4^ However, our results refine this concept by suggesting that tau deposition exerts its pathological effects through local deposition, and remotely, through network‐based disruption. In other words, the cognitive consequences of tau accumulation are not solely a function of regional protein aggregation but are mediated by its impact on neuronal function.

A more detailed mechanistic understanding of how tau pathology translates into both local and remote neuronal dysfunction may inform the development of novel biomarkers for therapeutic monitoring. In particular, individual patterns of cerebral hypoperfusion could serve as a functional readout to evaluate the efficacy of tau‐targeting interventions and their impact on clinical progression. In addition, remote neuronal dysfunction—unlike irreversible neuronal loss—may be potentially reversible, similar to the concept of diaschisis.^45^ This raises the possibility that interventions targeting the functional consequences of tau pathology, rather than tau itself, could help restore perfusion and alleviate cognitive deficits in regions affected through network‐mediated mechanisms.

A major strength of our study is the simultaneous assessment of tau deposition and cerebral hypoperfusion within a single imaging session, thereby reducing inter‐scan physiological and technical variability. We have previously demonstrated that perfusion estimates derived from dynamic [^18^F]PI‐2620 PET closely correspond to established markers of neuronal metabolism, including FDG PET or early‐phase amyloid PET, overall showing the feasibility of dynamic [^18^F]PI‐2620 to assess neuronal dysfunction and tau deposition in a single session.^22^, ^25^

However, several limitations should also be noted regarding the interpretation of our results: A spatial mismatch between cerebral hypoperfusion and tau deposition could be attributed to additional co‐pathologies frequently occurring in AD such as cerebral amyloid angiopathy (CAA), TDP‐43, or Lewy body pathology.^46^, ^47^, ^48^ Moreover, limited sensitivity of current tau PET tracers to detect early, subthreshold tau accumulation may lead to a relative overestimation of remote compared to local tau‐related effects on neuronal dysfunction. Importantly, in line with the “two‐hit” hypothesis of AD, vascular dysfunction itself may act as an upstream contributor to tau accumulation, raising the possibility that hypoperfusion could, at least in part, promote rather than solely result from tau pathology.^49^ However, given the cross‐sectional design of the present study, our findings do not allow us to determine the temporal sequence between vascular dysfunction and tau accumulation, and longitudinal studies will be required to disentangle whether hypoperfusion primarily precedes, follows, or co‐evolves with tau pathology. Furthermore, we used resting‐state functional MRI as a marker of connectivity, which is inferred based on shared inter‐regional blood oxygen level–dependent signal fluctuations and substantially, though not entirely, overlaps with structural connectivity assessed via diffusion MRI.^50^ We selected functional connectivity because tau spread is thought to be activity dependent and functional connectivity captures the multi‐synaptic connections that may underlie remote neuronal dysfunction not resolved by diffusion imaging.^51^, ^52^, ^53^ Structural connectivity has shown similar associations with tau spread and provides the backbone for functional connections, but as restricting functional to structurally connected regions previously left these associations unchanged, we retained functional connectivity as our primary metric.^26^

In conclusion, the present study provides converging evidence that neuronal dysfunction in AD is not solely a consequence of local tau accumulation but reflects a connectivity‐constrained process shaped by the network architecture of cortical tau epicenters. By integrating tau PET, perfusion PET, and functional connectivity, we demonstrate that hypoperfusion follows network proximity to tau epicenters across the disease continuum, arising in regions remote from tau when pathology is still spatially restricted and increasingly converging with local tau burden as biological disease severity progresses. These findings support a stage‐dependent model in which tau pathology initially induces remote, network‐mediated dysfunction before evolving into predominantly local neurotoxic damage. Furthermore, the observed mediation of the tau–cognition relationship by hypoperfusion underscores neuronal dysfunction as a critical intermediate mechanism linking molecular pathology to clinical decline. Taken together, our results refine current models of AD progression by highlighting the interplay between network topology and regional tau burden in shaping brain‐wide dysfunction, and suggest that functional imaging markers of hypoperfusion may serve as sensitive readouts of tau effects, therapeutic response, or resilience to tau‐induced neuronal dysfunction.

## CONFLICT OF INTEREST STATEMENT

J.V. received consulting fees, speaker fees, research support, and/or coverage for conference and travel expenses from Biogen, Eisai, Lilly, Novo Nordisk, the Alzheimer's Association, the Austrian Alzheimer's Society, the German Society of Nuclear Medicine, Helmholtz, and LMUexcellent. R.W. reports speaker honoraria from Novartis. N.F. received consulting, speaker, or travel honoraria from MSD, Life Molecular Imaging, Lantheus, GE Healthcare, Eisai, Eli Lilly, and Biogen and research support from Avid Radiopharmaceuticals. All other authors report no competing interests. Author disclosures are available in the Supporting Information.

## CONSENT STATEMENT

All study participants whose data are used in this study provided written informed consent.

## Supporting information




**Supporting Information**: alz71914‐sup‐0001‐SuppMat.docx

**Supporting Information**: alz71914‐sup‐0002‐ICJME.pdf
