# Supplementary material for: Network‐dependent remote effects and local tau burden jointly shape neuronal dysfunction in Alzheimer's disease
Source: Alzheimers Dement. 2026 Oct 7;22(10):e71914. doi: 10.1002/alz.71914 (PMC13644913; doi:10.1002/alz.71914)
Supplement: Supplementary file 2 [file ALZ-22-e71914-s002.docx]

**Supplementary materials**

**Network-Dependent Remote Effects and Local Tau Burden Jointly Shape Neuronal Dysfunction in Alzheimer’s Disease**

Lukas Frontzkowski^1,2^, Sebastian Roemer-Cassiano^1,3^, Johannes Gnörich^2^, Daniel Janowitz^1^, Hannah Eyob^2^, Katharina Probst^2^, Dario Dhoqina^2^, Anna Dewenter^1^, Anna Steward^1^, Zeyu Zhu^1^, Madleen Klonowski^1^, Fabian Hirsch^1^, Davina Biel^1^, Melina Paulus^1^, Jonathan Vöglein^3^, Sonja Schönecker^3^, Boris-Stephan Rauchmann^4,5^, Carolin Kurz^4^, Carolin Koriath^4^, Jan Häckert^6^, Sophia Stöcklein^7^, Carla Palleis^3^, Robert Perneczky^4,8,9,10,11^, Johannes Levin^3,8,9^, Katharina Bürger^1^, Rudolf A. Werner^2,12^, Günter Höglinger^3,8,9^, Matthias Brendel^2,8,9*^, Nicolai Franzmeier^1,9,13*^

* contributed equally

1. Institute for Stroke and Dementia Research (ISD), LMU University Hospital, Ludwig-Maximilians-University (LMU) Munich, Germany
2. Department of Nuclear Medicine, LMU University Hospital, Ludwig-Maximilians-University (LMU) Munich, Germany
3. Department of Neurology, LMU University Hospital, Ludwig-Maximilians-University (LMU) Munich, Germany
4. Department of Psychiatry and Psychotherapy, LMU University Hospital, Ludwig-Maximilians-University (LMU) Munich, Germany
5. Department of Neuroradiology, LMU University Hospital, Ludwig-Maximilians-University (LMU) Munich, Germany
6. Department of Psychiatry, Psychotherapy and Psychosomatics, Medical Faculty, University of Augsburg, BKH Augsburg, Augsburg, Germany.
7. Department of Radiology, LMU University Hospital, Ludwig-Maximilians-University (LMU) Munich, Germany
8. German Center for Neurodegenerative Diseases (DZNE) Munich, Munich, Germany.
9. Munich Cluster for Systems Neurology (SyNergy), Munich, Germany
10. Ageing Epidemiology (AGE) Research Unit, School of Public Health, Imperial College London, Fulham Palace Road, London, W6 8RP, UK.
11. Sheffield Institute for Translational Neuroscience (SITraN), University of Sheffield, 385a Glossop Road, Sheffield, S10 2HQ, UK.
12. The Russell H. Morgan Department of Radiology and Radiological Sciences, Division of Nuclear Medicine, Johns Hopkins School of Medicine, Baltimore, Maryland, USA.
13. University of Gothenburg, The Sahlgrenska Academy, Institute of Neuroscience and Physiology, Department of Psychiatry and Neurochemistry, Mölndal and Gothenburg, Sweden

**Figure 1**: Group-average [^18^F]PI-2620 perfusion (R1) and 30-60min [^18^F]PI-2620 SUVR z-score images rendered on cortical surfaces for each subgroup. Kinetic modeling was used to estimate perfusion parameters (R1). The inferior cerebellar grey matter (GM) served as reference tissue for both image modalities. SUVR - Standardized Uptake Value Ratio. SCD - Subjective Cognitive Decline, MCI - Mild Cognitive Impairment, DEM - Dementia.

**Figure 2**: Group-level tau epicenters derived from the top 10% Schaefer Rois with the highest mean [^18^F]PI-2620 DVR z-scores. The top-10% tau epicenters were located predominantly in bilateral posterior cortex: the superior and inferior parietal lobules, temporo-parietal junction, precuneus/posterior cingulate, lateral temporal, and extrastriate occipital cortex.

**Figure 3**: (**A**) Scatterplot depicting the group-level spatial association between cerebral perfusion (R1) and 30-60min [^18^F]PI-2620 SUVR based on the Schaefer200 cortical atlas. The conventional inferior cerebellar grey matter served as reference tissue. (**B**) Group-level spatial association between cerebral perfusion (R1) and connectivity-based distance to tau epicenters as scatterplot. (**C**) Distribution of the subject-level standardized β coefficients quantifying the spatial association of cerebral perfusion with local tau deposition (pastel blue) and with connectivity-based distance to tau epicenters (pastel pink) across all 131 patients. Each dot represents one patient; violins show the full distribution, boxes the median and interquartile range, and diamonds the group mean. The β and P values above each distribution denote the mean standardized β and the corresponding one-sample t-test against zero. Pastel pink - Spatial association between connectivity-based distance to tau epicenters and perfusion. SUVR - Standardized Uptake Value Ratio.

**Figure 4**: (**A**) Scatterplot illustrating the individual effect sizes between [^18^F]PI-2620 tau-PET and perfusion (R1) as a function of the global spatial extent of tau deposition (proportion of tau-PET positive - i.e. > 1.5) voxels relative to the total numbers of brain voxels) (**B**) Scatterplot illustrating the individual effect sizes between connectivity-based distance to tau epicenters and perfusion as a function of the global spatial extent of tau deposition. (**C**) Spatial dissocation between regional hypoperfusion and tau deposition illustrated for three groups of AD patients. AD patients were assigned to three groups based on the amount of spatial tau extent. Surface renderings illustrate the total number of AD patients that show a perfusion-tau mismatch in a given region of interest (ROI). Spatial pathology mismatch is higher in groups with low (Q1) or medium (Q2) spatial tau extent with a predominant localization in the parietal and temporal cortex in Q1, shifting towards frontal regions and the cingulum in Q2 (red). In AD patients with the highest global tau extent (Q3), spatial mismatch was largely absent, reflexting a near complete overlap between tau deposition and cerebral perfusion (blue). Q1 - Low spatial extent of tau pathology; Q2 - medium spatial extent of tau pathology, Q3 - high spatial extent of tau pathology. SUVR - Standardized Uptake Value Ratio.
